# IgE Mediated allergy to wheat in a child with celiac disease

**DOI:** 10.1186/1710-1492-10-S1-A37

**Published:** 2014-03-03

**Authors:** Tiffany Wong, Edmond S Chan

**Affiliations:** 1 Department of Pediatrics, Division of Allergy & Immunology, Faculty of Medicine, University of British Columbia, Vancouver, BC, Canada

## Introduction

Celiac disease and immediate type hypersensitivity to wheat are immune responses with different pathogenic mechanisms. Both diseases are well known entities but their coexistence in the same patient is rarely reported.

## Case presentation

We report the case of a girl with celiac disease who subsequently developed IgE mediated hypersensitivity to wheat. The patient is a Caucasian female who was diagnosed with celiac disease at 18 months of age after presenting with recurrent vomiting and failure to thrive. Her anti-tTG antibody level was greater than 200 E.U. and biopsy results from endoscopy were consistent with celiac disease. Specific IgE antibody to wheat was negative at 2 years of age. Around seven years of age, she developed immediate symptoms of urticaria, cough and shortness of breath with accidental exposures to wheat. Specific IgE antibody testing was repeated and positive to wheat (42.5 kU/L), as well as rye (33.9 kU/L), barley (53.4 kU/L) and oat (11.3kU/L). At 9 years of age, skin prick testing was positive to wheat, barley and rye but negative to oat. The patient has subsequently tolerated an open oral food challenge to oat. She continues to avoid wheat, rye and barley and carries an epinephrine autoinjector at all times.

## Conclusion

To our knowledge, this is the first report of a patient with celiac disease and concomitant IgE-mediated allergy to wheat presenting with immediate symptoms in two body systems. Although the pathophysiology of these diseases is different, this case demonstrates that they are not exclusive of one another. In clinical assessment of celiac disease over time, development of IgE mediated allergy is possible and should be considered.

